# Treatment with Antiangiogenic Drugs in Multiple Lines in Patients with Metastatic Colorectal Cancer: Meta-Analysis of Randomized Trials

**DOI:** 10.1155/2016/9189483

**Published:** 2016-08-30

**Authors:** R.-D. Hofheinz, U. Ronellenfitsch, S. Kubicka, A. Falcone, I. Burkholder, U. T. Hacker

**Affiliations:** ^1^Interdisziplinäres Tumorzentrum, Universitätsmedizin Mannheim, University of Heidelberg, Mannheim, Germany; ^2^Department of Surgery, Universitätsmedizin Mannheim, University of Heidelberg, Mannheim, Germany; ^3^Krebszentrum Reutlingen, Reutlingen, Germany; ^4^University of Pisa, Pisa, Italy; ^5^Department of Nursing and Health, University of Applied Sciences of the Saarland, Saarbrücken, Germany; ^6^University Cancer Center Leipzig (UCCL), Universitätsklinikum Leipzig, Leipzig, Germany

## Abstract

*Background*. In metastatic colorectal cancer (mCRC), continuing antiangiogenic drugs beyond progression might provide clinical benefit. We synthesized the available evidence in a meta-analysis.* Patients and Methods*. We conducted a meta-analysis of studies investigating the use of antiangiogenic drugs beyond progression. Eligible studies were randomized phase II/III trials. Primary endpoints were overall survival (OS) and progression-free survival (PFS). Secondary endpoints were the impact of continuing antiangiogenic drugs (i) in subgroups, (ii) in different types of compounds targeting the VEGF-axis (monoclonal antibodies versus tyrosine kinase inhibitors), and (iii) on remission rates and prevention of progression.* Results*. Eight studies (3,668 patients) were included. Continuing antiangiogenic treatment beyond progression significantly improved PFS (HR 0.64; 95%-CI, 0.55–0.75) and OS (HR 0.83; 95%-CI, 0.76–0.89). PFS was significantly improved in all subgroups with comparable HR. OS was improved in all subgroups stratified by age, gender, and ECOG status. The rate of patients achieving at least stable disease was improved with an OR of 2.25 (95%-CI, 1.41–3.58).* Conclusions*. This analysis shows a significant PFS and OS benefit as well as a benefit regarding disease stabilization when using antiangiogenic drugs beyond progression in mCRC. Future studies should focus on the optimal sequence of administering antiangiogenic drugs.

## 1. Introduction

First-line chemotherapy for metastatic colorectal cancer (mCRC) is frequently combined with antiangiogenic agents, namely, bevacizumab, a monoclonal antibody targeting vascular endothelial growth factor (VEGF) A. This is based on the results of several studies demonstrating a benefit in progression-free survival [1–4]. However, a statistically significant prolongation of overall survival has only been observed in one of these first-line studies [2] raising the question how long the optimum duration of the use of antiangiogenic drugs should be.

Prolonged duration of bevacizumab treatment* until* definitive progression has been shown to improve progression-free survival in the phase 3 NO16966 trial which investigated the addition of bevacizumab to oxaliplatin-based first-line regimens [3]. Moreover, two observational studies showed a correlation between the use of bevacizumab* beyond* progression and improved overall survival in advanced colorectal cancer [5, 6]. Preclinical data strongly suggest that continued antiangiogenic treatment beyond progression might provide antitumor efficacy even in further lines of treatment [7, 8].

These observations led to the setup of clinical trials, which investigated the use of bevacizumab beyond progression in patients who had undergone a bevacizumab-based first-line treatment. Two trials (TML [9] and BEBYP [10, 11]), although using different inclusion criteria and endpoints, unequivocally demonstrated that the continued use of bevacizumab beyond progression improved PFS (TML and BEBYP) and overall survival (TML).

More recently, randomized trials investigated other drugs with antiangiogenic properties in second and further lines of treatment in patients with metastatic colorectal cancer after pretreatment with bevacizumab-based regimens. For instance, the phase-3 VELOUR trial investigated the addition of aflibercept (a fusion protein trapping VEGF-A, VEGF-B, and placental growth factor [PlGF]) in combination with 5-fluorouracil and irinotecan (FOLFIRI) treatment in patients who had been pretreated with oxaliplatin-based regimens [12]. It was demonstrated that the addition of aflibercept improved progression-free and overall survival. Notably, this also held true for the patients with bevacizumab-pretreatment [13]. The concept of continued use of antiangiogenic drugs has also been demonstrated in the CORRECT study and the CONCUR study in which treatment with regorafenib—a multikinase inhibitor targeting among others VEGF-receptor 2—was superior to best supportive care in patients who had been pretreated with all active drugs including bevacizumab [14, 15]. Taken together, data from recent studies suggest that prolonged duration of antiangiogenic treatment might be associated with improved outcome in patients with metastatic colorectal cancer.

In the present meta-analysis we sought to investigate the concept of treatment with antiangiogenic drugs in multiple lines beyond progression by analyzing aggregate data of randomized trials. Special emphasis was given on describing potential improvements of progression-free and overall survival related to specific subgroups including KRAS wildtype patients.

## 2. Patients and Methods

### 2.1. Objectives of Meta-Analysis and Eligibility Criteria

Primary objective of the present analysis was to investigate progression-free survival (PFS) and overall survival (OS) in patients with metastatic colorectal cancer who had been pretreated with an antiangiogenic treatment and underwent antiangiogenic treatment beyond progression.

Secondary objectives were to assess the effects of the continued or repeated antiangiogenic treatment in subgroups (stratified by age, sex, ECOG status, and tumor KRAS mutational status) and in studies using anti-VEGF treatment (i.e., bevacizumab and aflibercept) versus tyrosine kinase inhibitors (TKI). Moreover, we investigated the response rates (i.e., the rate of evaluable patients achieving complete or partial remissions) and the rate of “tumor stabilization,” that is, the rate of evaluable patients without primary progression while receiving treatment.

Only randomized phase II and III trials were included in the current meta-analysis. The inclusion of subgroups of randomized trials was allowed provided sufficient information was given in the trial reports. Only studies performed with the approval of an appropriate ethics committee and conducted in compliance with the declaration of Helsinki were included in this meta-analysis.

Antiangiogenic treatment was defined as the use of drugs targeting at least one important angiogenic pathway, for instance, monoclonal antibodies targeting VEGF or VEGF-receptors, or (multi)TKI targeting angiogenic pathways.

### 2.2. Information Sources, Search Strategy, and Study Selection

Searches in PubMed and proceedings of international meetings were conducted to identify studies with information relevant for the current analysis.

Eligible studies were phase II or III, randomized, controlled trials comparing (i) antiangiogenic drugs in combination with either active treatment (i.e., chemotherapy) or placebo with (ii) active treatment or placebo alone in patients who had previously been treated with antiangiogenic drugs for metastatic colorectal cancer. We used MeSH and full-text search terms for metastatic colorectal cancer and molecular targeted therapies, limiting our results to English language articles published in PubMed between January 1, 2007, and October 11, 2015. For PubMed, the search was ((((“molecular targeted therapy” [All Fields] OR (“molecular” [All Fields] AND “targeted” [All Fields]) AND (“therapy” [All Fields] OR “therapies” [All Fields]) AND (“colorectal neoplasms” [All Fields] OR “colorectal cancer” [All Fields]) OR (“colorectal” [All Fields] AND “cancer” [All Fields]) AND (“randomized” [All Fields] OR “randomized study” [All Fields]) AND English [lang])))).

In addition to computerized search, references of retrieved papers were also screened for missing trials.

To minimize publication bias we conducted a manual search of conference abstracts. For conferences, the search was “colorectal cancer” or “advanced colorectal cancer”, manually limited to abstracts on targeted therapies. The proceedings of the following meetings were examined for presented abstracts limiting the search to the years 2007–2016: (i) American Society of Clinical Oncology (ASCO) annual meetings; (ii) ASCO Gastrointestinal Cancer Symposium; (iii) European Society for Medical Oncology (EMSO) and European multidisciplinary cancer congress (ECCO) meetings; (iv) World Congress on Gastrointestinal Cancer. Two independent reviewers (RDH, UR, or UH) assessed title, keywords, and abstracts of retrieved studies. If studies met the inclusion criteria, they assessed full texts and mutually decided on inclusion.

### 2.3. Data Collection

Data extraction was conducted independently by three investigators (RDH, UR, and UH) in accordance with the Preferred Reporting Items for Systematic Reviews and Meta-Analyses (PRISMA) guidance [19]. For each study the following information was extracted: publication or presentation date, first author's last name, sample size/sample size of subgroup of interest, primary endpoints, information pertaining to study design, PFS, and OS as well as response definition, regimens used, line of treatment, number of outcome events, data on PFS, OS, response to treatment, and data of subgroups of interest.

### 2.4. Synthesis of Results, Statistical Methods, and Analyses

The impact of antiangiogenic treatment on overall survival (OS) and progression-free survival (PFS) was measured in terms of the hazard ratio (HR). In all studies included in this meta-analysis, the HR was calculated as ratio of the active therapy divided by the hazard of the control group. Therefore, a HR lower than 1 indicates a benefit of the active treatment, whereas a HR higher than 1 indicates a higher risk of death or progression of the active treatment, respectively. The estimates of the HR were extracted directly from the publications and variance of the estimates was calculated from published confidence intervals of the HR. For the TML study, unstratified as well as stratified HRs were reported. As the unstratified analysis was the primary analysis of this study, the unstratified estimates were included in the meta-analysis.

Heterogeneity of the individual HRs was tested using Cochran's *Q* statistic [19]. If heterogeneity was not detected at the 10% significance level, the fixed effects model was used. If the test for heterogeneity was significant, the overall HR was calculated using the random effects model [20]. Heterogeneity was quantified by *I* ^2^ coefficient measuring the percentage of total variation across studies that is due to heterogeneity rather than by chance.

Meta-analysis of the remission, progression, and rates of “tumor stabilization” was performed using the same statistical methods described above. In the remission analysis and in the progression analysis patients with unknown status were counted as progressive. Finally, in the “prevention of progression” (POP) analysis patients with unknown status were excluded.

ML-estimates of the odds ratio (OR) were determined from reported contingency tables and the within-trial variance was computed from the inverse of the matrix of second derivatives of the log-likelihood (Woolf's formula).

## 3. Results

### 3.1. Selected Trials

The Medline search was done on June 15, 2016. It resulted in 3,252 articles. Hand-searches of conference proceedings were conducted including all conferences until June 2016.

**Figure 1:**
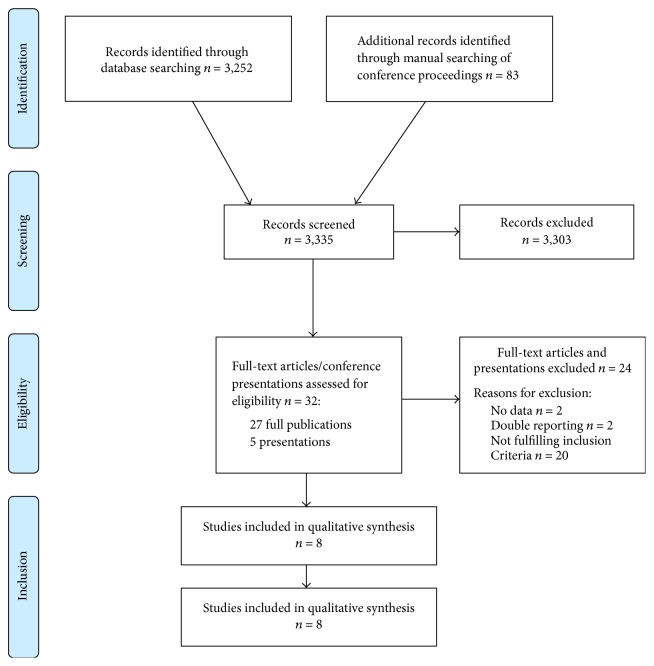
PRISMA diagram.

Based on the criteria defined above the following eight studies were selected for inclusion into the current meta-analysis (PRISMA diagram; cf.  Figure 1): TML trial [9], BEBYP trial [10, 11], subgroup of VELOUR study [12], CORRECT trial [14], subgroup of AGITG CO.20 trial [16], a subgroup of patients treated within a randomized phase II trial (FOSCO) investigating the addition of sorafenib to second-line chemotherapy [17], subgroups of the CONCUR study [15], and the RAISE study [18].

Trial characteristics are depicted in Table 1. All trials were evaluable for PFS and OS.

Table 2 shows the number of patients evaluable from respective trials for the determination of primary and secondary outcome parameters.

### 3.2. Combined Analysis: Primary Outcomes—Progression-Free and Overall Survival

**Figure 2:**
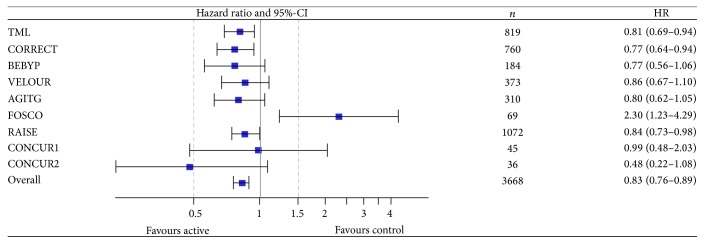
Meta-analysis for overall survival. CI: confidence interval; HR: hazard ratio.

**Figure 3:**
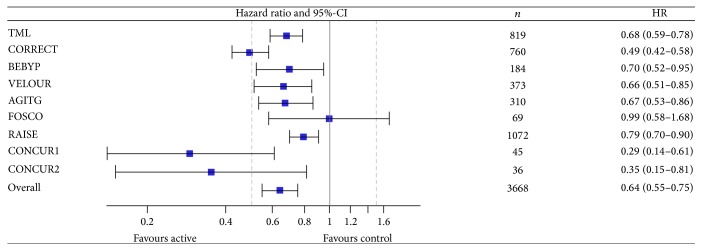
Meta-analysis for progression-free survival. CI: Confidence interval; HR: hazard ratio.

The use of antiangiogenic drugs beyond progression improved progression-free survival and overall survival over control (i.e., best supportive care or active treatment alone). The hazard ratio for overall survival (*n* = 3,668) was 0.83 (95%-CI, 0.76–0.89); (Figure 2). The test for heterogeneity was significant (*p* = 0.010; *I* ^2^ = 40%). PFS was improved (*n* = 3,668) with a HR of 0.64 (95%-CI, 0.55–0.75); test for heterogeneity: *p* < 0.001; *I* ^2^ = 74%; (Figure 3).

### 3.3. Combined Analysis: Secondary Outcomes—Subgroup Analyses of Progression-Free and Overall Survival

The results of the subgroup analyses for PFS and OS according to sex, age, ECOG status, and KRAS status (WT versus MUT) are given in Table 3 (PFS) and Table 4 (OS) and in Supplementary Figures S1–S8 in Supplementary Material available online at http://dx.doi.org/10.1155/2016/9189483. In all subgroup analyses (age, using a cut-off of 65 years, ECOG performance status, gender, and tumor KRAS mutational status) the use of antiangiogenic drugs beyond progression improved PFS with comparable hazard ratios in both dichotomized groups, respectively. Similarly, overall survival results are comparable for dichotomized subgroups regarding age and ECOG performance status. However, the benefit of using antiangiogenic drugs beyond progression regarding OS was weaker in the subgroup of women (*n* = 1,047) with a HR of 0.81 (95%-CI, 0.70–0.94) and absent in patients bearing a tumor with KRAS mutation (*n* = 1,260) with a HR of 0.89; 95%-CI, 0.78–1.02.

PFS was improved both in studies using mAB targeting the VEGF-axis (*n* = 2,448), HR 0.73 (95%-CI, 0.67–0.79) (no heterogeneity was identified *p* = 0.37, *I* ^2^ = 0%), and in studies using TKI (*n* = 1,220), HR 0.55 (95%-CI, 0.38–0.78) (test for heterogeneity *p* = 0.010, *I* ^2^ = 70.%) (Supplementary Figure S9). The pooled HR for OS regarding trials investigating monoclonal antibodies (mAB) targeting the VEGF-axis (TML, BEBYP, VELOUR subgroup, and RAISE; *n* = 2448) was 0.83 (95%-CI, 0.75–0.91) (test for heterogeneity *p* = 0.93, *I* ^2^ = 0%) and the pooled HR for OS in studies investigating the use of TKI beyond progression (CORRECT, FOSCO, AGITG subgroup, and CONCUR subgroups; *n* = 1220) was 0.92 (95%-CI, 0.59–1.42) (test for heterogeneity *p* = 0.011, *I* ^2^ = 69.%) (Supplementary Figure S10).

**Figure 4:**
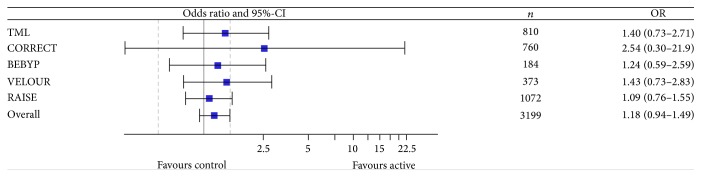
Meta-analysis of remission rate. CI: confidence interval; OR: odds ratio.

**Figure 5:**
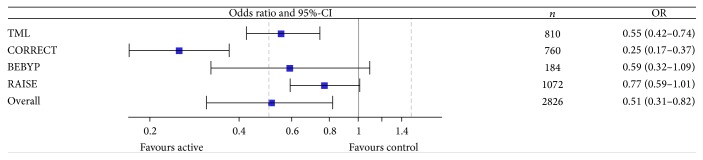
Meta-analysis of progression rate. CI: confidence interval; OR: odds ratio.

**Figure 6:**
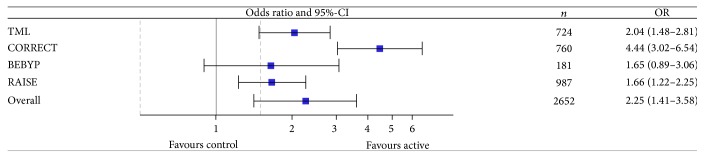
Meta-analysis of prevention of progression rate. CI: confidence interval; OR: odds ratio.

The use of antiangiogenic drugs beyond progression did not increase response rates (*n* = 5 trials included in meta-analysis with a total of *n* = 3,199 patients) (Table 5 and Figure 4). The odds ratio (OR) was 1.18 (95%-CI, 0.94–1.49). In contrast, progression rate was decreased (*n* = 4 trials included in meta-analysis with a total of *n* = 2,826 patients) (Table 6 and Figure 5). The odds ratio (OR) was 0.51 (95%-CI, 0.31–0.82). Finally, the rate of evaluable patients achieving at least stable disease (*n* = 4 trials reporting data with a total of *n* = 2,652 patients) was improved with an OR of 2.25 (95%-CI, 1.41–3.58) (Table 7 and Figure 6).

## 4. Discussion

Using aggregate data from eight randomized trials we found a clinically relevant and significant improvement of PFS and OS for the use of antiangiogenic drugs beyond progression with a cumulative hazard ratio of 0.64 for PFS and 0.83 for OS. The test for heterogeneity of study results was significant for both OS and PFS. The latter was mainly influenced by the results of the FOSCO trial for PFS and OS and additionally of the CORRECT trial for PFS. While the relative risk reduction for PFS was 35%, the survival benefit was 17% (HR 0.83). The main outlier in the OS analysis was the small FOSCO trial, which, however, had a relatively strong impact on the hazard ratio. Excluding FOSCO from the meta-analysis would result in an even lower hazard ratio underlining a significant benefit of continuing antiangiogenic drugs after first progression. FOSCO is one of a couple of studies investigating the addition of multityrosine kinase inhibitors to a chemotherapy doublet. All of these trials—regardless of the treatment setting, that is, 1st or 2nd line—have resulted in negative study results mainly caused by increased toxicity, decreased dose intensity, and compromised quality of life (e.g., CONFIRM or HORIZON studies). Thus, with the exception of FOSCO we found relatively homogeneous results regarding the patient-relevant endpoint of overall survival with a relative risk reduction for death of approximately 20% for continuation of antiangiogenic treatment beyond progression. Interestingly, the effects of different concepts (i.e., continuing bevacizumab beyond progression or switching to other antiangiogenic agents, for instance, aflibercept or ramucirumab) resulted in comparable hazard ratios for OS ranging between 0.77 and 0.86. Therefore, either approach might be considered for patients progressing after bevacizumab-based first-line therapy and the decision should be made in light of toxicity, patient preference, and drug approval status.

We also investigated the effects of continued antiangiogenic treatment in subgroups. The decision to carry out analyses stratified by age, ECOG status, and gender as well as KRAS mutational status was mainly taken due to the fact that other subgroups of interest (for instance, liver only metastases and time interval between last bevacizumab treatment) have not been reported by a sufficiently high number of trials to enable a meta-analysis. In all subgroup analyses an improved PFS was found. The HR of the respective dichotomized groups (age <65 versus ≥65 years; women versus men, ECOG 0 versus ≥1, and KRAS wildtype versus KRAS mutation) were comparable. Similarly, overall survival results are comparable for dichotomized subgroups regarding age and ECOG performance status. However, the effect of using antiangiogenic drugs beyond progression regarding OS was weaker in the subgroup of women (*n* = 1,047) with a HR of 0.81 (95%-CI, 0.70–0.94) and not statistically significant for patients bearing a tumor with KRAS mutation (*n* = 1,260) with a HR of 0.89 (95%-CI, 0.78–1.02). The latter finding is difficult to interpret. However, a significant PFS benefit was demonstrated for both tumors with KRAS wildtype and KRAS mutations (HR 0.60 and 0.66, resp.) which suggests that patients with RAS mutations should not be excluded from continued treatment with antiangiogenic agents.

No improvement in response rates was seen; however, the rate of progression was decreased. Additionally, we analyzed the potential to prevent tumor progression (defined as the number of patients with evaluable remission status achieving at least stable disease). In this analysis a clinically relevant benefit for the use of antiangiogenic drugs beyond progression was found. This is in line with earlier findings from the first-line setting, indicating that the activity of bevacizumab in combination with chemotherapy with respect to a prolongation of PFS is predominantly driven by disease stabilization [21].

In all, the current meta-analysis demonstrates the usefulness of continued antiangiogenic drugs beyond progression regarding their potential to improve PFS and OS in a clinically meaningful manner. The benefit was seen in the subgroups stratified by age, gender, and ECOG performance status.

Limitations of the current analysis are that no individual patient data were used and toxicity could not be assessed. Furthermore, although the antiangiogenic activity of the drugs included in the analysis may differ, we decided to include studies with multityrosine kinase inhibitors such as regorafenib as well, because these drugs are believed to exert their main activity via antiangiogenic mechanisms. Moreover, the size of some subgroups assessed in our analyses was small. Therefore, statistical power of single analyses might have been too low to show a significant difference in these subgroups, namely, women and KRAS-mutant tumors. Our meta-analysis, synthesizing data from several trials, indicates that using antiangiogenic drugs beyond and after progression can meanwhile be regarded as an established strategy in the treatment of patients with metastatic colorectal cancer. Future research should especially focus on the optimal sequence of using these antiangiogenic drugs, for instance, the timing of the switch from bevacizumab to broader active drugs such as aflibercept or regorafenib. Clearly, in this aspect biomarkers will be needed to elucidate if inhibition of alternative angiogenic pathways or additional tyrosine kinases would be required for continued antiangiogenic activity or if the patient could remain on bevacizumab treatment.

## Supplementary Material

Figures S1 and S2: Metaanalyses for progression-free and overall survival according to age. *CI*: Confidence interval; *hr*: Hazard ratio; yrs: years
Figures S3 and S4: Metaanalyses for progression-free and overall survival according to gender. *CI*: Confidence interval; *hr*: Hazard ratio; yrs: years
Figures S5 and S6: Metaanalyses for progression-free and overall survival according to Eastern Cooperative Oncology Group Performance (ECOG) status. *CI*: Confidence interval; *hr*: Hazard ratio; 0: ECOG 0; >=1: ECOG status ≥ 1
Figures S7 and S8: Metaanalyses for progression-free and overall survival according to KRAS mutational status. *CI*: Confidence interval; *hr*: Hazard ratio; WT: KRAS wildtype tumor; mutated: tumor harboring KRAS mutation
Figures S9 and S10: Metaanalyses for progression-free and overall survival according to substance class used (monoclonal antibody, mAB, tyrosine kinase inhibitor, TKI). *CI*: Confidence interval; *hr*: Hazard ratio.

## Figures and Tables

**Table 1:** Overview of studies included in meta-analysis.

Study	First author/year	Investigational treatment	Control treatment	Primary endpoint	Number of patients (ITT) (active/control)
TML	Bennouna 2012 [9]	Chemotherapy (oxaliplatin or irinotecan based) plus bevacizumab	Chemotherapy (oxaliplatin or irinotecan based) alone	OS	819 (409/410)
CORRECT	Grothey 2013 [14]	Regorafenib	Placebo	OS	760 (505/255)
BEBYP	Masi 2013 [10]	Chemotherapy (FOLFIRI, mFOLFOX) plus bevacizumab	Chemotherapy (FOLFIRI, mFOLFOX) alone	PFS	184 (92/92)
VELOUR subgroup	Allegra 2012 [13]	FOLFIRI plus aflibercept	FOLFIRI	OS	373 (186/187)
AGITG subgroup	Siu 2013 [16]	Cetuximab plus brivanib	Cetuximab	OS	310 (152/158)
FOSCO	Hoehler 2013 [17]	FOLFOX/FOLFIRI plus sorafenib	FOLFOX/FOLFIRI plus placebo	PFS	69 (32/37)
RAISE	Tabernero 2015 [18]	FOLFIRI plus ramucirumab	FOLFIRI plus placebo	PFS	1072 (536/536)
CONCUR	Li 2015 [15]	Regorafenib	Placebo	OS	81

**Table 2:** Overview of number of studies and patients available for primary and secondary analyses.

	Progression-free survival	Overall survival
*Primary endpoint*	*n* = 8 trials (*n* = 3,688 pts)	*n* = 8 trials (*n* = 3,668 pts)

*Secondary endpoint: PFS/OS subgroup analyses*		
Gender (male/female)	*n* = 4 trials (*n* = 3,017 pts)	*n* = 3 trials (*n* = 2,651 pts)
Age (cut-off 65 years)	*n* = 4 trials (*n* = 2,835 pts)	*n* = 3 trials (*n* = 2,651 pts)
ECOG status (0 versus ≥1)	*n* = 4 trials (*n* = 2,825 pts)	*n* = 3 trials (*n* = 2,641 pts)
KRAS status (WT/MUT)	*n* = 4 trials (*n* = 2,545 pts)	*n* = 3 trials (*n* = 2,417 pts)

*Secondary endpoint: remission/progression analyses*		
Remission rate	*n* = 5 trials (*n* = 3,199 pts)	
Progression rate	*n* = 4 trials (*n* = 2,826 pts)	
Prevention of progression rate	*n* = 4 trials (*n* = 2,652 pts)	

**Table 3:** Results of subgroup analyses for progression-free survival.

Subgroup	*n*	HR	95% CI	Test of heterogeneity *Q*-value	Test of heterogeneity *p* value	*I* ^2^ coefficient
Age						
<65 years	1670	0.62	0.47–0.82	*Q* = 27.7950	*p* < 0.001	89.21%
≥65 years	1165	0.74	0.66–0.84	*Q* = 2.1756	*p* = 0.54	0%

Sex						
Male	1907	0.68	0.54–0.84	*Q* = 13.2772	*p* = 0.004	77.40%
Female	1110	0.66	0.49–0.89	*Q* = 15.3943	*p* = 0.002	80.51%

ECOG status						
0	1440	0.61	0.48–0.80	*Q* = 16.3356	*p* < 0.001	81.64%
≥1	1385	0.73	0.60–0.88	*Q* = 6.5342	*p* = 0.088	54.09%

KRAS status						
Wildtype	1219	0.60	0.47–0.76	*Q* = 14.7202	*p* = 0.02	76.62%
Mutation	1326	0.66	0.53–0.83	*Q* = 15.5233	*p* = 0.001	80.67%

**Table 4:** Results of subgroup analyses for overall survival.

Subgroup	*n*	HR	95% CI	Test of heterogeneity *Q*-value	Test of heterogeneity *p* value	*I* ^2^ coefficient
Age						
<65 years	1578	0.80	0.71–0.90	*Q* = 1.2902	*p* = 0.52	0%
≥65 years	1073	0.84	0.73–0.97	*Q* = 0.0354	*p* = 0.98	0%

Sex						
Male	1604	0.82	0.73–0.92	*Q* = 3.8902	*p* = 0.14	48.59%
Female	1047	0.81	0.70–0.94	*Q* = 3.4071	*p* = 0.18	41.30%

ECOG status						
0	1290	0.76	0.67–0.88	*Q* = 0.9274	*p* = 0.63	0%
≥1	1351	0.86	0.76–0.97	*Q* = 0.7502	*p* = 0.69	0%

KRAS status						
Wildtype	1157	0.74	0.64–0.85	*Q* = 1.7867	*p* = 0.41	0%
Mutation	1260	0.89	0.78–1.02	*Q* = 0.09216	*p* = 0.95	0%

**Table 5:** Analysis of response rate. Number of patients achieving tumor remissions according to treatment.

Study name	Active treatment		Control		OR	95%-CI
	Remission	No remission	Remission	No remission		
TML	22	382	16	390	1.40	[0.73–2.71]
CORRECT	5	500	1	254	2.54	[0.30–21.86]
BEBYP	19	73	16	76	1.24	[0.59–2.59]
VELOUR	22	164	16	171	1.43	[0.73–2.83]
RAISE	72	464	67	469	1.09	[0.76–1.55]

Overall	Test of heterogeneity: *Q* = 1.2927, *p* = 0.86, *I* ^2^ = 0%				**1.18**	[**0.94–1.49]**

**Table 6:** Analysis of tumor progression. Number of patients experiencing tumor progression according to treatment.

Study name	Active treatment		Control		OR	95%-CI
	Progression	No progression	Progression	No progression		
TML	129	275	186	220	0.55	[0.42–0.74]
CORRECT	298	207	217	38	0.25	[0.17–0.37]
BEBYP	28	64	39	53	0.59	[0.32–1.09]
RAISE	139	397	167	369	0.77	[0.59–1.01]

Overall	Test of heterogeneity: *Q* = 21.9488, *p* < 0.001, *I* ^2^ = 86.33% → random effect model				**0.51**	[**0.31–0.82]**

**Table 7:** Analysis of tumor stabilization rate. Number of patients experiencing at least stable disease according to treatment.

Study name	Active treatment		Control		OR	95%-CI
	No progression	Progression	No progression	Progression		
TML	275	87	220	142	2.04	[1.48–2.81]
CORRECT	221	284	38	217	4.44	[3.02–6.54]
BEBYP	64	27	53	37	1.65	[0.89–3.06]
RAISE	397	87	369	134	1.66	[1.22–2.25]

Overall	Test of heterogeneity: *Q* = 17.03610, *p* < 0.001, *I* ^2^ = 82.39% → random effect model				2.25	[1.41–3.58]
