# Supplementary material for: Treatment with Antiangiogenic Drugs in Multiple Lines in Patients with Metastatic Colorectal Cancer: Meta-Analysis of Randomized Trials
Source: Gastroenterol Res Pract. 2016 Aug 30;2016:9189483. doi: 10.1155/2016/9189483 (PMC5021498; doi:10.1155/2016/9189483)
Supplement: Supplementary file 1 — Figures S1 and S2: Metaanalyses for progression-free and overall survival according to age. CI: Confidence interval; hr: Hazard ratio; yrs: years Figures S3 and S4: Metaanalyses for progression-free and overall survival according to gender. CI: Confidence interval; hr: Hazard ratio; yrs: years Figures S5 and S6: Metaanalyses for progression-free and overall survival according to Eastern Cooperative Oncology Group Performance (ECOG) status. CI: Confidence interval; hr: Hazard ratio; 0: ECOG 0; >=1: ECOG status ≥ 1 Figures S7 and S8: Metaanalyses for progression-free and overall survival according to KRAS mutational status. CI: Confidence interval; hr: Hazard ratio; WT: KRAS wildtype tumor; mutated: tumor harboring KRAS mutation Figures S9 and S10: Metaanalyses for progression-free and overall survival according to substance class used (monoclonal antibody, mAB, tyrosine kinase inhibitor, TKI). CI: Confidence interval; hr: Hazard ratio. [file 9189483.f1.docx]

**Figures**

**Figures S1 and S2:** Metaanalyses for progression-free and overall survival according to age. *CI*: Confidence interval; *hr*: Hazard ratio; yrs: years

***Figure S1 – PFS***


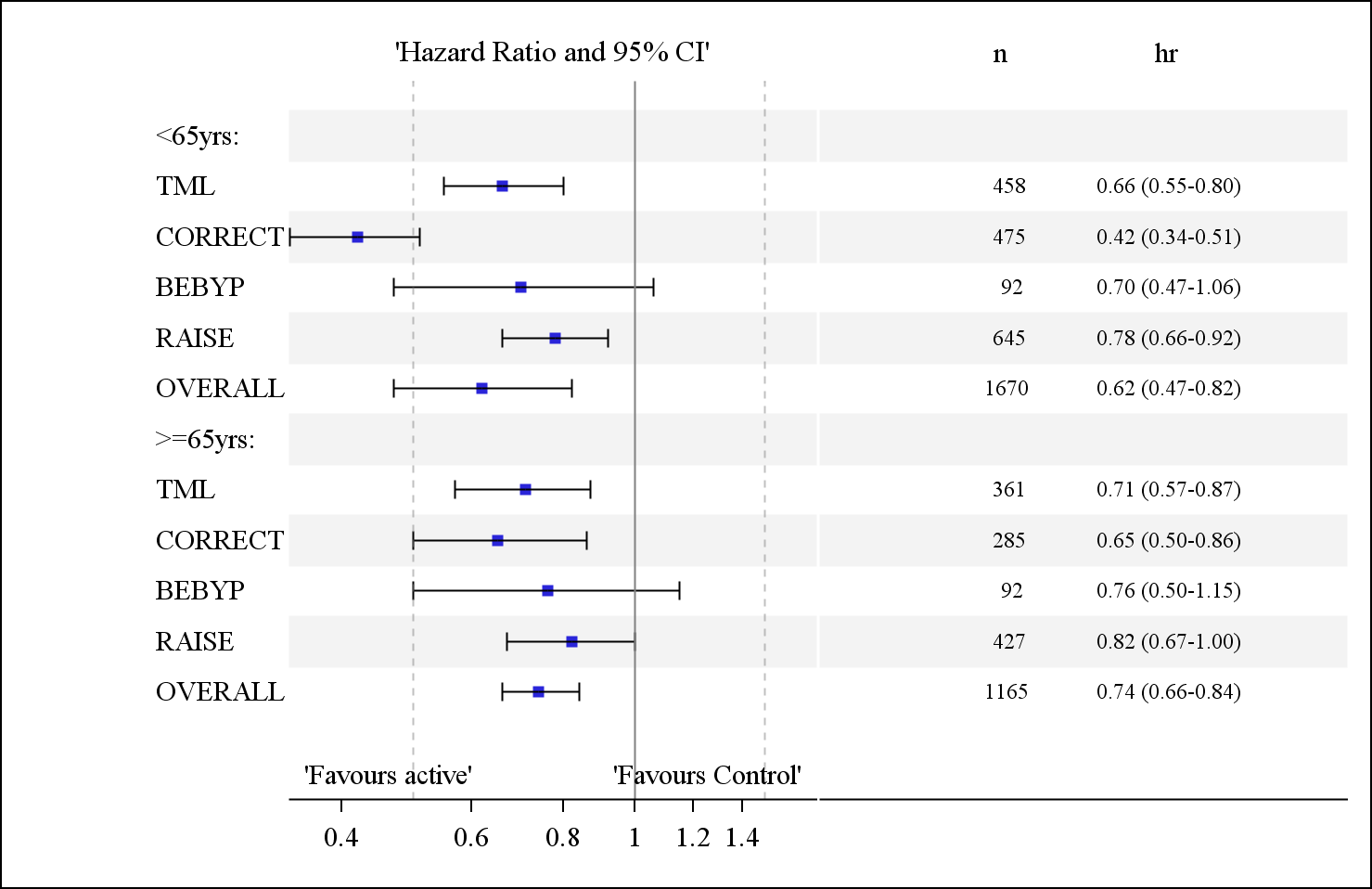


**Note:** CIs for BEBYP were extracted from forest plot published in Masi et al., 2015

***Figure S2 – OS***


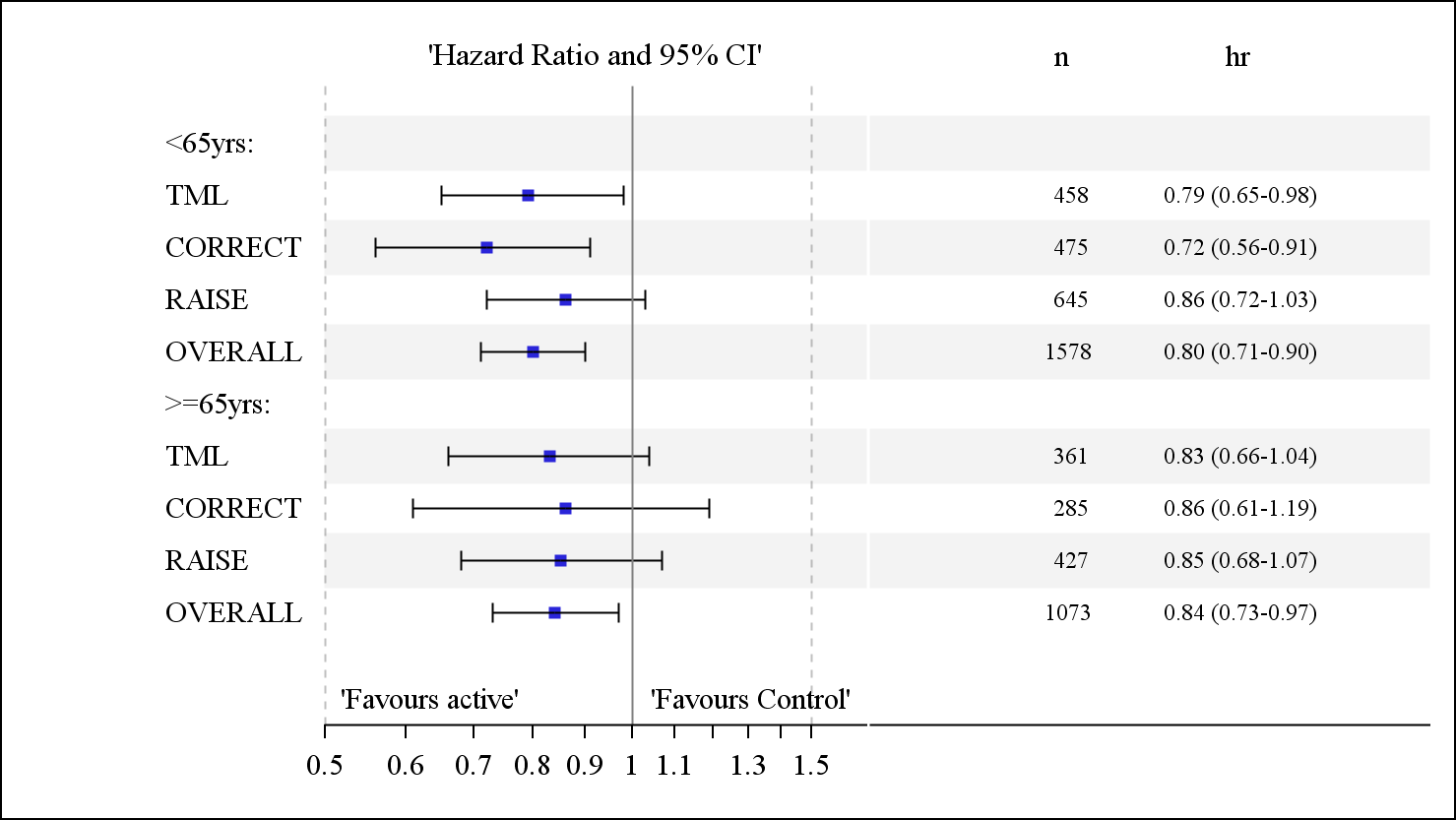


**Note:** CIs for BEBYP were extracted from forest plot published in Masi et al., 2015

**Figures S3 and S4:** Metaanalyses for progression-free and overall survival according to gender. *CI*: Confidence interval; *hr*: Hazard ratio; yrs: years

***Figure S3 – PFS***


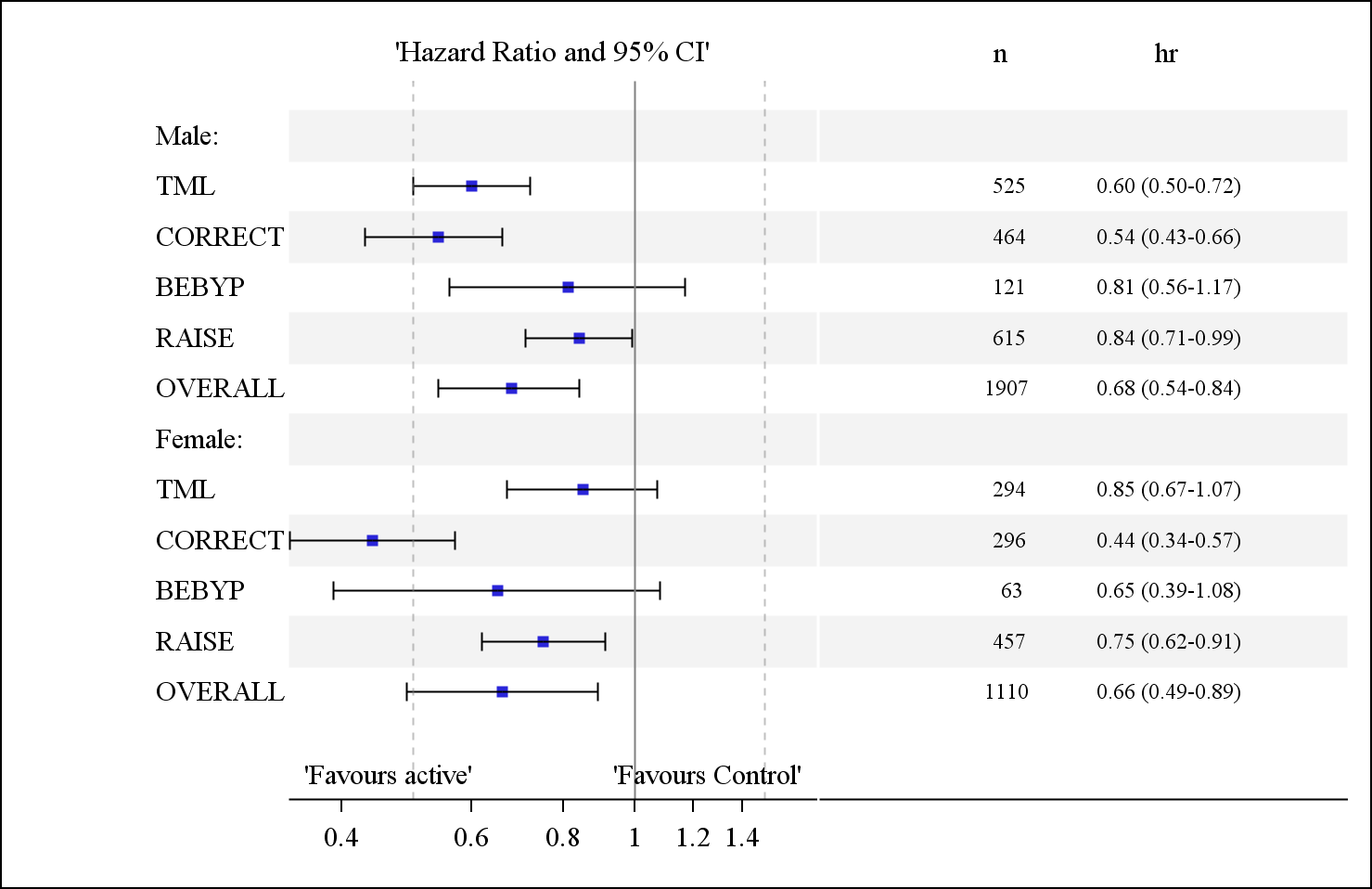


**Note:** CIs for BEBYP were extracted from forest plot published in Masi et al., 2015

***Figure S4 – OS***


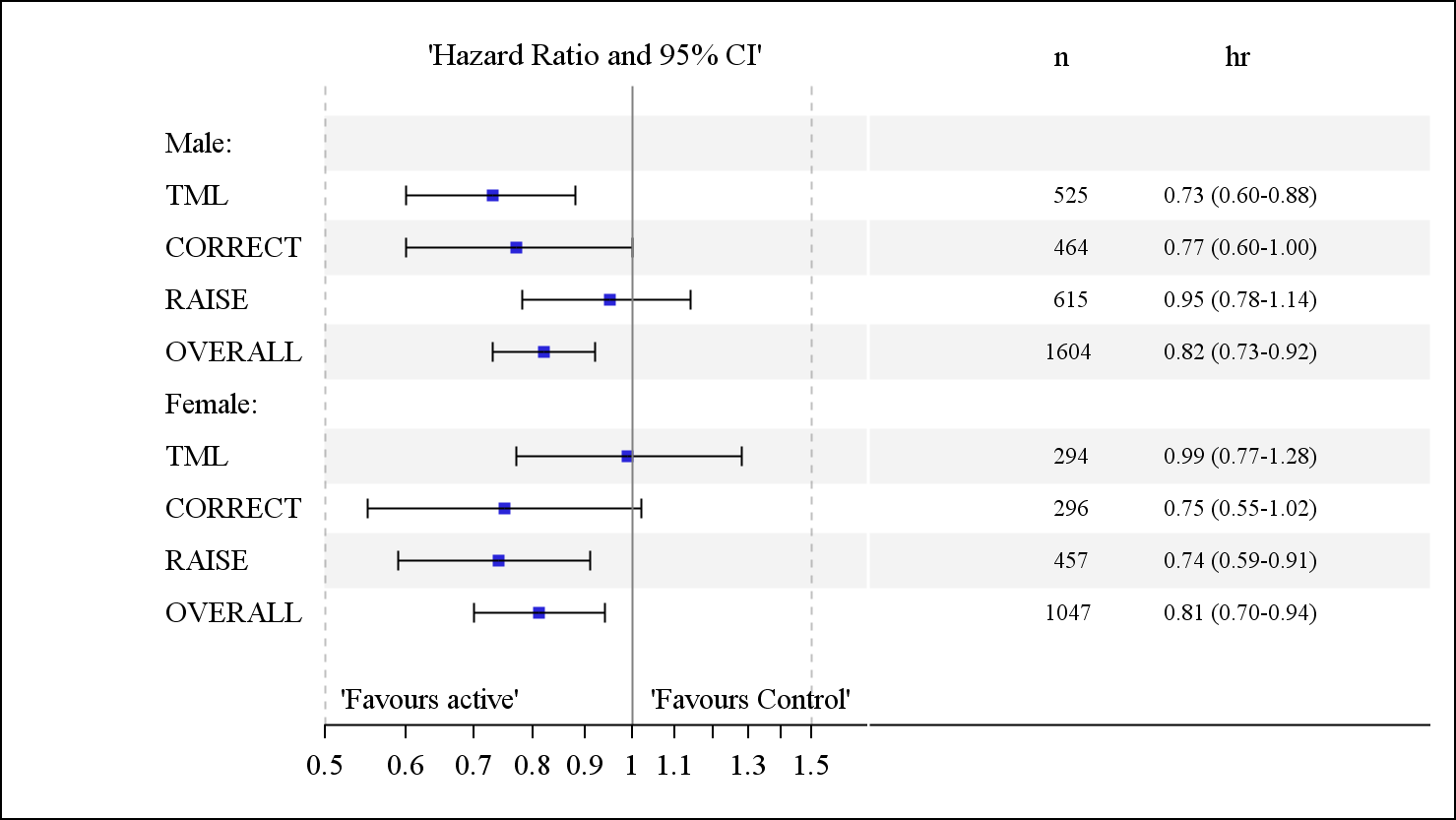


**Note:** CIs for BEBYP were extracted from forest plot published in Masi et al., 2015

**Figures S5 and S6:** Metaanalyses for progression-free and overall survival according to Eastern Cooperative Oncology Group Performance (ECOG) status. *CI*: Confidence interval; *hr*: Hazard ratio; 0: ECOG 0; >=1: ECOG status ≥ 1

***Figure S5 – PFS***


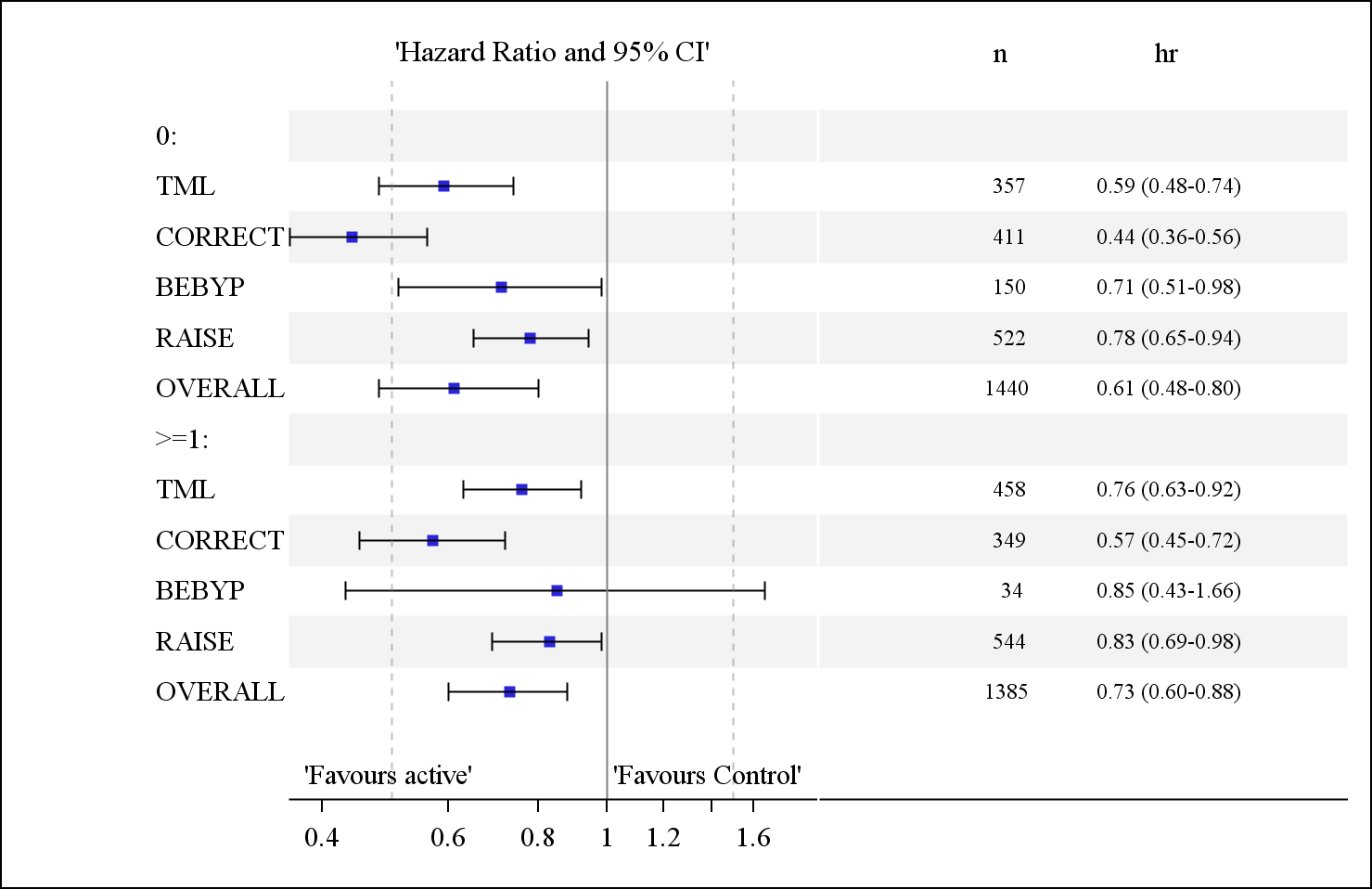


**Note:** CIs for BEBYP were extracted from forest plot published in Masi et al., 2015

***Figure S6 – OS***


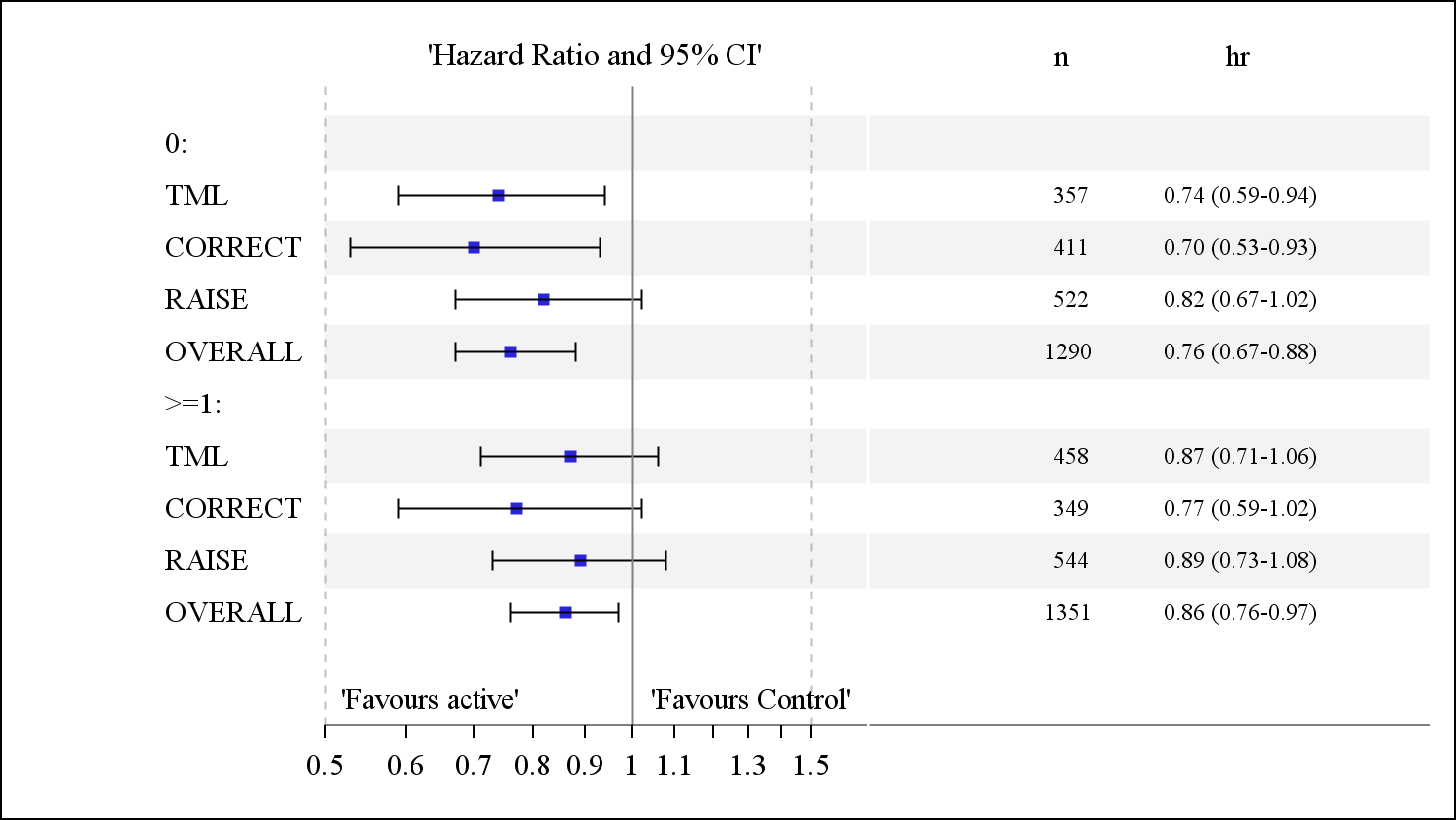


**Figures S7 and S8:** Metaanalyses for progression-free and overall survival according to KRAS mutational status. *CI*: Confidence interval; *hr*: Hazard ratio; WT: KRAS wildtype tumor; mutated: tumor harboring KRAS mutation

***Figure S7 – PFS***


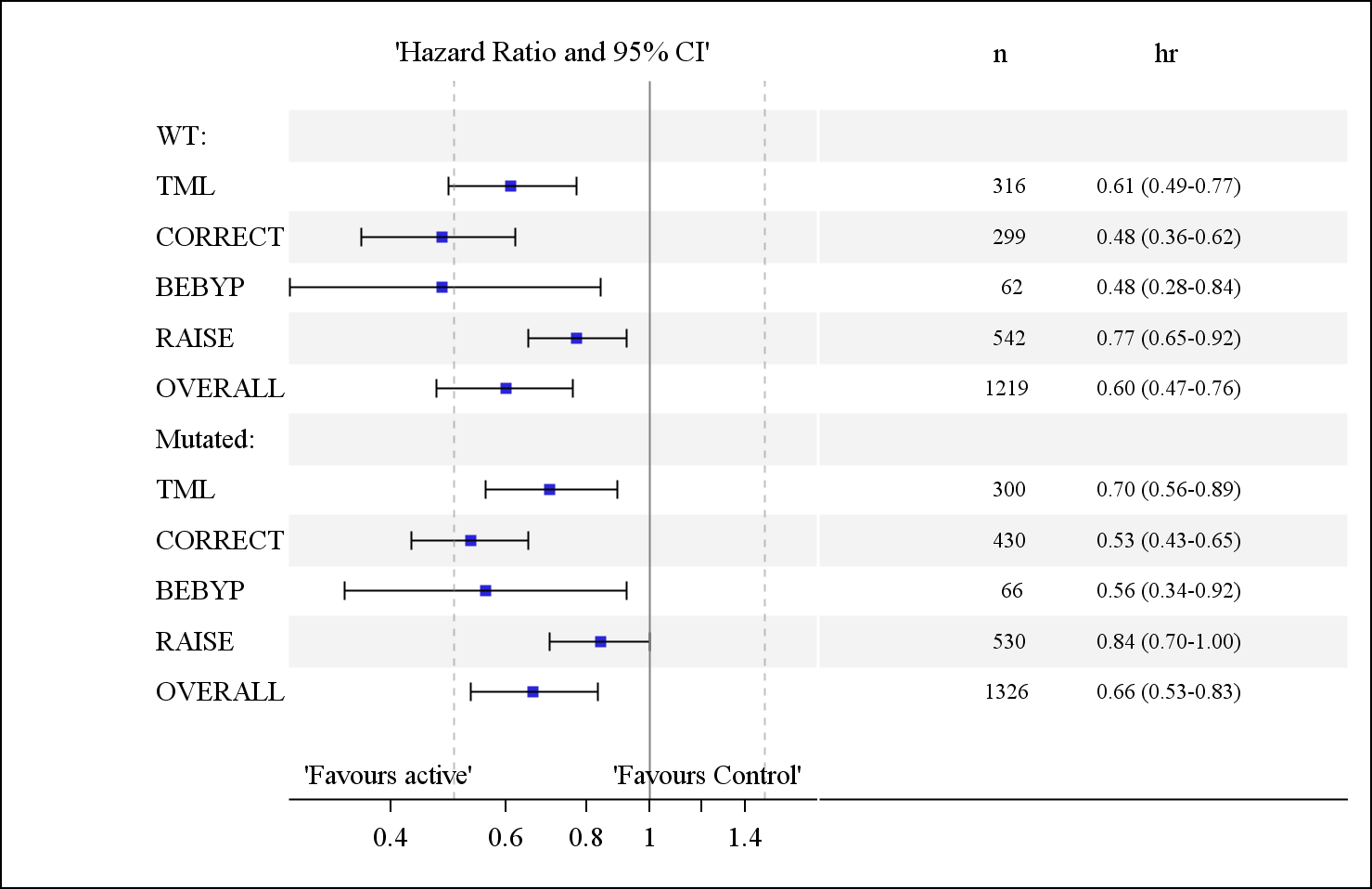


***Figure S8 – OS***


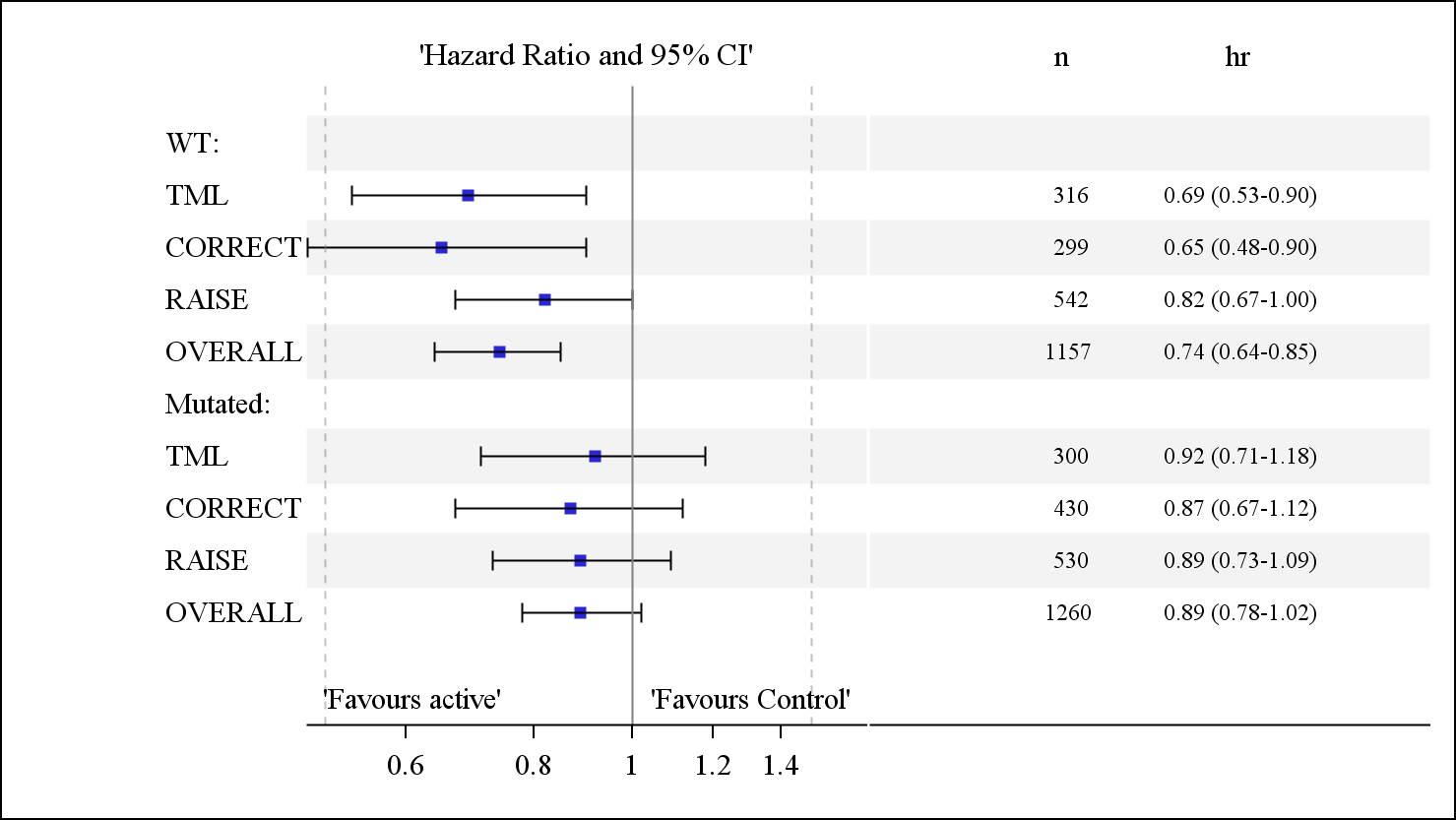


**Figures S9 and S9:** Metaanalyses for progression-free and overall survival according to compound (mAB trageting VEGF-axis vs. TKI). *CI*: Confidence interval; *hr*: Hazard ratio;

***Figure S9 – PFS***


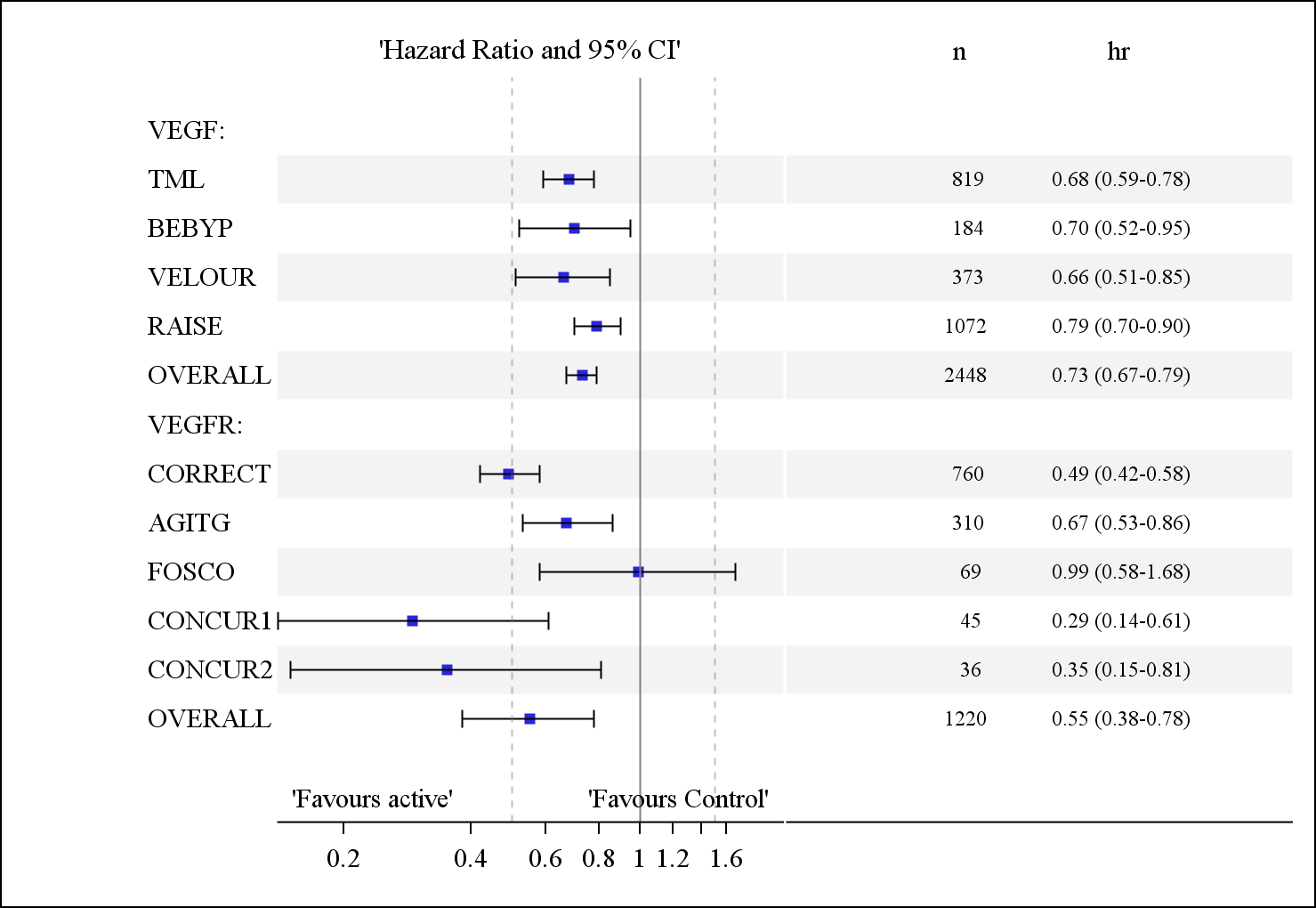


mAB

TKI

**Note:** CI for FOSCO reported as 90%CI was transformed into a 95% CI

CONCUR^1^:previous anti-VEG but no previous anti-EGFR treatment
CONCUR^2^:previous anti-VEG and previous anti-EGFR treatment

***Figure S10 –OS***


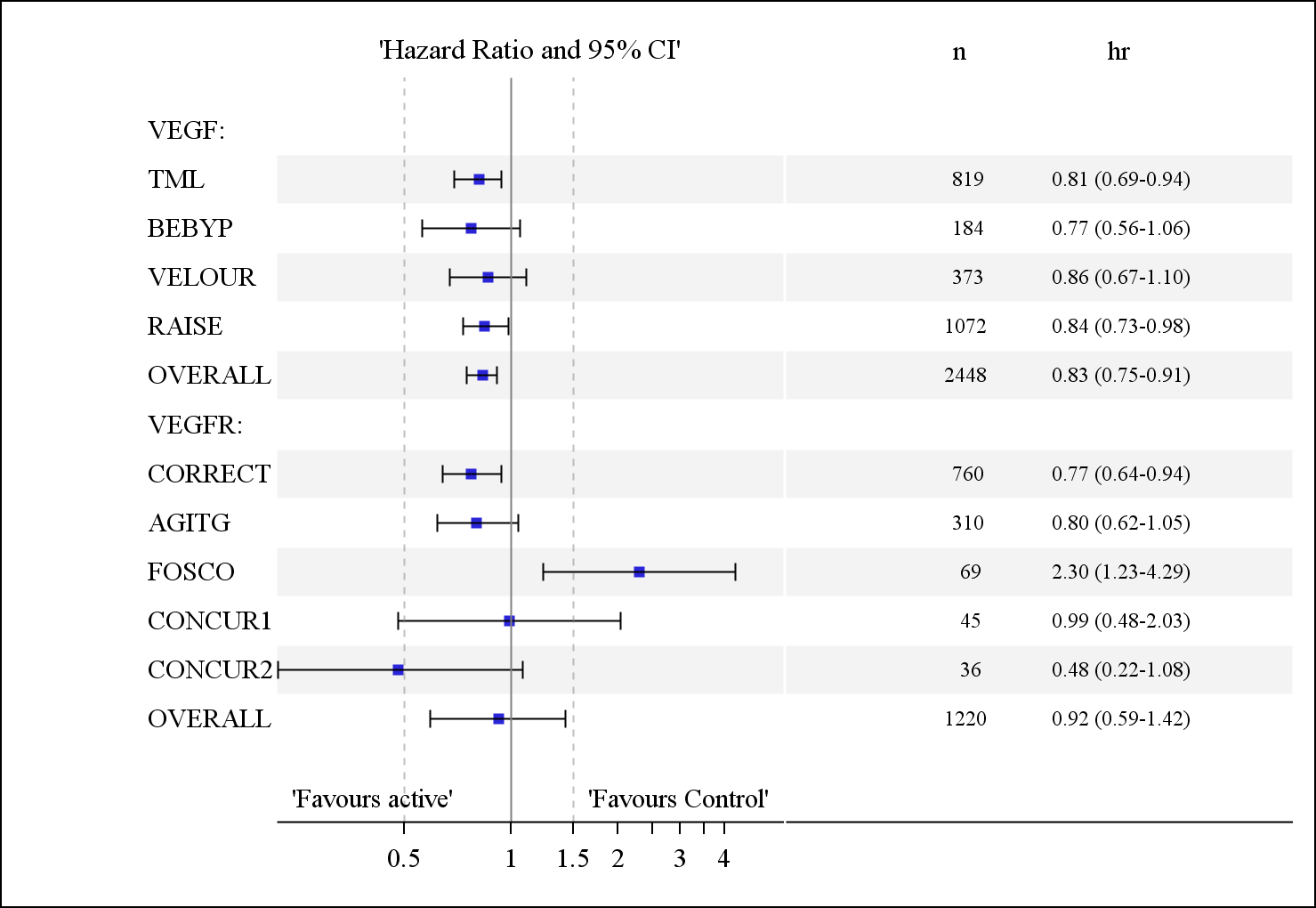


mAB

TKI

**Note:** CI for FOSCO reported as 90%CI was transformed into a 95% CI

CONCUR^1^:previous anti-VEG but no previous anti-EGFR treatment
CONCUR^2^:previous anti-VEG and previous anti-EGFR treatment
